# Altered functional connectivity strength between structurally and functionally affected brain regions in visual snow syndrome

**DOI:** 10.1093/braincomms/fcaf171

**Published:** 2025-05-05

**Authors:** Myrte Strik, Meaghan J Clough, Emma J Solly, Rebecca Glarin, Owen B White, Scott C Kolbe, Joanne Fielding

**Affiliations:** Spinoza Centre for Neuroimaging, Netherlands Institute for Neuroscience, Royal Netherlands Academy for Arts and Sciences (KNAW), 1105 BA Amsterdam, the Netherlands; Computational Cognitive Neuroscience and Neuroimaging, Netherlands Institute for Neuroscience, Royal Netherlands Academy for Arts and Sciences (KNAW), 1105 BA Amsterdam, the Netherlands; Melbourne Brain Centre Imaging Unit, Department of Radiology, Melbourne Medical School, University of Melbourne, Parkville, VIC 3010, Australia; Department of Neuroscience, Central Clinical School, Monash University, Melbourne, VIC 3004, Australia; Department of Neuroscience, Central Clinical School, Monash University, Melbourne, VIC 3004, Australia; Melbourne Brain Centre Imaging Unit, Department of Radiology, Melbourne Medical School, University of Melbourne, Parkville, VIC 3010, Australia; Department of Neuroscience, Central Clinical School, Monash University, Melbourne, VIC 3004, Australia; ARC Centre of Excellence in Optical Microcombs for Breakthrough Science, Royal Melbourne Institute of Technology University, Melbourne, VIC 3000, Australia; Department of Neuroscience, Central Clinical School, Monash University, Melbourne, VIC 3004, Australia

**Keywords:** visual snow syndrome, functional connectivity, fMRI, 7T, resting-state MRI

## Abstract

Visual snow syndrome (VSS) is a neurological disorder that is predominantly characterized by persistent, dynamic visual disturbances, experienced across the entire visual field. Earlier research highlighted the significance of distinct brain regions, exhibiting alterations in both anatomical structure and functional characteristics. To further investigate the functional role of these regions, we examined the resting-state connectivity between these areas in individuals with VSS and the relation with VSS symptoms and oculomotor measures of visual processing. Forty patients with VSS (53% females; age = 33.2 ± 10.1 years; 22 with migraine) and 60 healthy controls (58% females; age = 32.0 ± 9.2 years) were scanned using 7 Tesla MRI system. High spatial and temporal resting-state (RS) functional (TR = 800 ms, 1.6 mm isotropic) and anatomical (MP2RAGE, 0.75 mm isotropic) images were acquired. Resting-state data were pre-processed (motion correction, temporal filtering and spatial smoothing), functional connectivity was calculated between regions of interest and compared between groups. Significant metrics were compared with VSS patients with and without migraine and correlated with oculomotor measures (prosaccade and anti-saccade latencies), number of VSS symptoms, self-rated VSS intensity and perceived disruptiveness. Compared to healthy controls, VSS patients demonstrated significantly higher connectivity between the supramarginal gyrus and lateral occipital cortex (*P* = 0.016) and fusiform (*P* = 0.007), lower connectivity between the supramarginal gyrus and pallidum (*P* = 0.032), as well as between the parahippocampal gyrus and lateral occipital cortex (*P* = 0.007), which related to higher perceived disruptiveness (*P* = 0.002, *r* = −0.489). No differences were found between VSS with and without migraine. This study revealed altered functional connectivity strength in individuals with VSS, suggesting stronger connectivity between cortical areas, particularly centred around the supramarginal gyrus, and disconnections with deep grey matter and temporal cortices, which associated with perceived disruptiveness of VSS.

## Teaser

Strik *et al*. found altered functional resting-state connectivity in people with visual snow syndrome, with increased cortical connectivity around the supramarginal gyrus and reduced connectivity with deep grey matter and temporal cortices, linked to the perceived disruptiveness of visual snow.

## Graphical Abstract

**Graphical Abstract:**
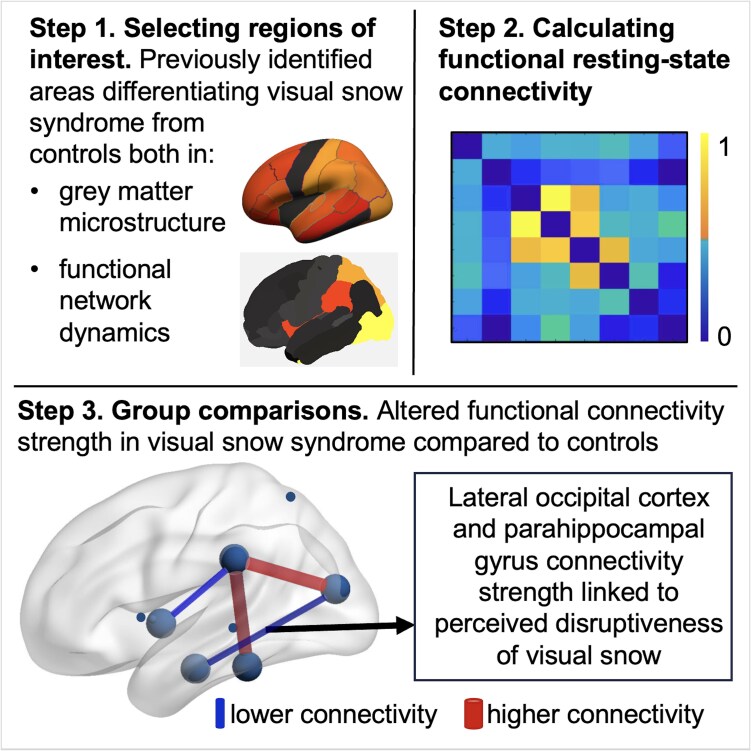

## Introduction

Visual snow syndrome (VSS) is a neurological condition characterized by continuous and dynamic visual disturbances across the entire visual field. Flickering dots are perceived with the eyes open and closed and are often described as black, white, or transparent. Besides the persistent presence of visual snow, the diagnostic criteria also require the manifestation of at least two other visual symptoms including trailing of moving objects (palinopsia), excessive floaters, moving bright dots (blue field entoptic phenomenon), self-lighting or spontaneous bright flashes of light (photopsia).^1^ Individuals with VSS can also experience a wide range of non-visual comorbidities. Most reported are auditory sensations (tinnitus) and migraine, alongside sensory sensations such as paraesthesia. Additionally, psychiatrics symptoms including depression, anxiety and depersonalization-derealization are also frequently observered.^2^ The combination of these persistent visual disturbances and non-visual symptoms can significantly impact the overall quality of daily life.^2,3^

MRI and behavioural ocular motor tasks have been used to understand the neuropathologic underpinnings of VSS, but the underlying mechanisms remain largely unknown. Where brain morphology differences are subtle,^4-7^ if evident in VSS compared to controls, the grey matter (GM) differences appear robust and widespread.^7^ To study the influence of microstructural involvement on brain function, functional MRI can be used. Previous research has shown that VSS patients demonstrate reduced activation in response to visual snow-like stimuli^8^ and widespread altered connectivity of areas within visual, attention and salience networks.^9,10^ These functional activity and connectivity changes appear either relatively focal and or subtle and, in some studies, shown clinically important.^8,9,11^ However, given the robust and widespread microstructural differences,^7^ rather than examining specific individual regions and isolated connections between those regions, we previously chose to first study the entire brain as a network.^11^ VSS brains were less variable over time in terms of network segregation and integration, centred around occipital and parietal cortices, potentially indicating an inflexible network structure.^11^ This altered network dynamics correlated to the typical oculomotor behaviour in VSS patients, i.e. faster responses towards a visual stimulus (prosaccade eye movements).^11,12^

To gain deeper understanding the underlying functional alterations and its relation to clinical symptoms of VSS, in this study, we decided to further study those regions that were affected both in terms of their anatomical structure as well as functional network characteristics.^7,11^ Rather than investigating the network dynamics, we assessed the connectivity strength between these anatomically and functionally relevant regions using high-resolution resting-state (RS) 7T MRI data. Given the altered microstructural and reduced functional network dynamics, we hypothesize that the connectivity strength will be altered, potentially centred around a specific region, and be clinically meaningful.

## Methods

### Participants

Forty patients with VSS (53% females; age = 33.2 ± 10.1 years; 22 with migraine) and 60 healthy controls (HC; 58% females; age = 32.0 ± 9.2 years) were included. All patients fulfilled the diagnostic VSS criteria as per the International Classification of Headache Disorders (http://ichd-3.org, A1.4.6). Equivalent numbers of patients experiencing migraine (*n* = 22; age = 34.7 ± 10.5 years, 15 with visual aura) and patients without a history of migraine (*n* = 17; age = 31.6 ± 9.7 years) were included. One patient did not self-report either the presence or absence of migraine. To minimize the impact of a migraine or migraine aura symptoms on the behavioural and MRI assessments, patients who experienced a migraine within 3 days before, during or up to 3 days after assessment were excluded. Additional exclusion criteria comprised of a history or presence of a neurological disease other than VSS, as well as potential confounding psychiatric or ophthalmological conditions. The latter was assessed using a full ophthalmic examination, retinal structure and function, colour vision and visual acuity.

### 7T MRI brain imaging session

Participants were scanned using a whole-body Siemens MAGNETOM 7 Tesla (7T) MRI system (Siemens Healthcare, Erlangen, Germany) with a combined single-channel transmit and 32-channel receive head coil (Nova Medical, Wilmington, MA, USA). High spatial and temporal functional imaging was acquired in an axial plane using a multiband multi-slice gradient echo, echo planar sequence.^3^ Participants were asked to fixate on a cross on the middle of the screen at the backend of the scanner, visible through a mirror. Functional MRI (fMRI) parameters included the following: repetition time = 800 ms, echo time = 22.2 ms, flip angle = 45 degrees, 84 slices, multiband factor=6, acceleration factor (AF) = 2, phase encoding direction = anterior-to-posterior, voxel size = 1.6 mm isotropic, image matrix = 130 × 130. As to anatomical images, high-resolution structural scans were acquired using a three-dimensional T1-weighted magnetization prepared 2 rapid acquisition gradient echoes (MP2RAGE) sequence with the following parameters: TR = 5000 ms, TE = 3.06 ms, inversion time = 700/2700 ms, FA = 4°/5°, 224 slices, AF = 4, PE = AP, voxel size = 0.75 mm isotropic, image matrix = 330 × 330, imaging plane = sagittal.

### Visual snow syndrome symptoms and visual processing

Participants were asked to rate the intensity and disruptiveness of their visual snow using Likert scales. VS intensity was rated on a 1–6 scale using an image displaying varying intensities of static as a guide, with 1 referring to the lowest intensity of static and 6 referring to the highest intensity. ‘Disruptiveness’ was rated on a 1–7 scale from 1 (‘Not at all disruptive’) to 7 (‘Severely disruptive’). The number of visual symptoms (in addition to VS) was also recorded. In addition, visual processing was assessed through two ocular motor tasks: the prosaccade (PS) and the anti-saccade (AS) task In the PS task, participants generated a saccade toward a suddenly appearing stimulus, while in the AS task, they directed the saccade to the mirror opposite location, more cognitively demanding.^12,13^ We identified an ocular motor profile in VSS patients, indicative of altered visual stimulus processing. Specifically, VSS patients generated faster responses to suddenly appearing visual stimulus (PS) and poorly suppressed a response to a suddenly appearing nontarget visual stimulus (AS). Results are described in detail in a previous publication.^12^

### Functional resting-state analyses

Resting-state fMRI data was pre-processed using MELODIC using standard settings (FSL5, FMRIB 2012, Oxford, UK, https://fsl.fmrib.ox.ac.uk/fsl/) including head motion correction (MCFLIRT), brain extraction (BET), spatial smoothing using double the voxel size (3.2 mm, full width at half-maximum Gaussian kernel) and temporal filtering (high-pass filter, 100 s cut-off). To account for global signal variations, further temporal filtering of each voxel signal time courses was performed involving linear regression of the mean white matter and cerebrospinal fluid time courses. To further minimize motion effects, additional motion correction steps were performed using FIX (FSL5, FMRIB 2012, Oxford, UK, https://fsl.fmrib.ox.ac.uk/fsl/). To train FIX, the independent components (ICA, MELODIC analyses) from a subset of subjects were used by manually identify components whose signal patterns were consistent with scan artefacts, physiological noise or head movement. These trained-weights files were then used to auto-classify components in the remaining subjects and to further improve and clean the data.

### Functional connectivity between regions of interest

We chose our regions of interest (ROI) based on our previous findings in VSS patients. In these recent publications, we observed widespread changes in cortical and subcortical GM microstructure, with the occipital cortices affected most profoundly, as well as functional network disturbances centred around the occipital and parietal lobules. We further investigated those regions affected both in terms of their structure and function, including the putamen, pallidum, fusiform, lateral occipital cortex, parahippocampal gyrus, banks of superior temporal sulcus, superior parietal cortex and supramarginal gyrus.

For each subject, these (sub)cortical regions were segmented using the T1-weighted MP2RAGE image and FreeSurfer (v.6.0-patch; https://surfer.nmr.mgh.harvard.edu/). The grey matter segmentations were manually edited if needed and registration to functional subject space was performed using the trans-formation matrices calculated from bbregister (FreeSurfer, v.6.0-patch) and FLIRT (FSL6, fsl.fmrib.ox.ac.uk).

**Figure 1:**
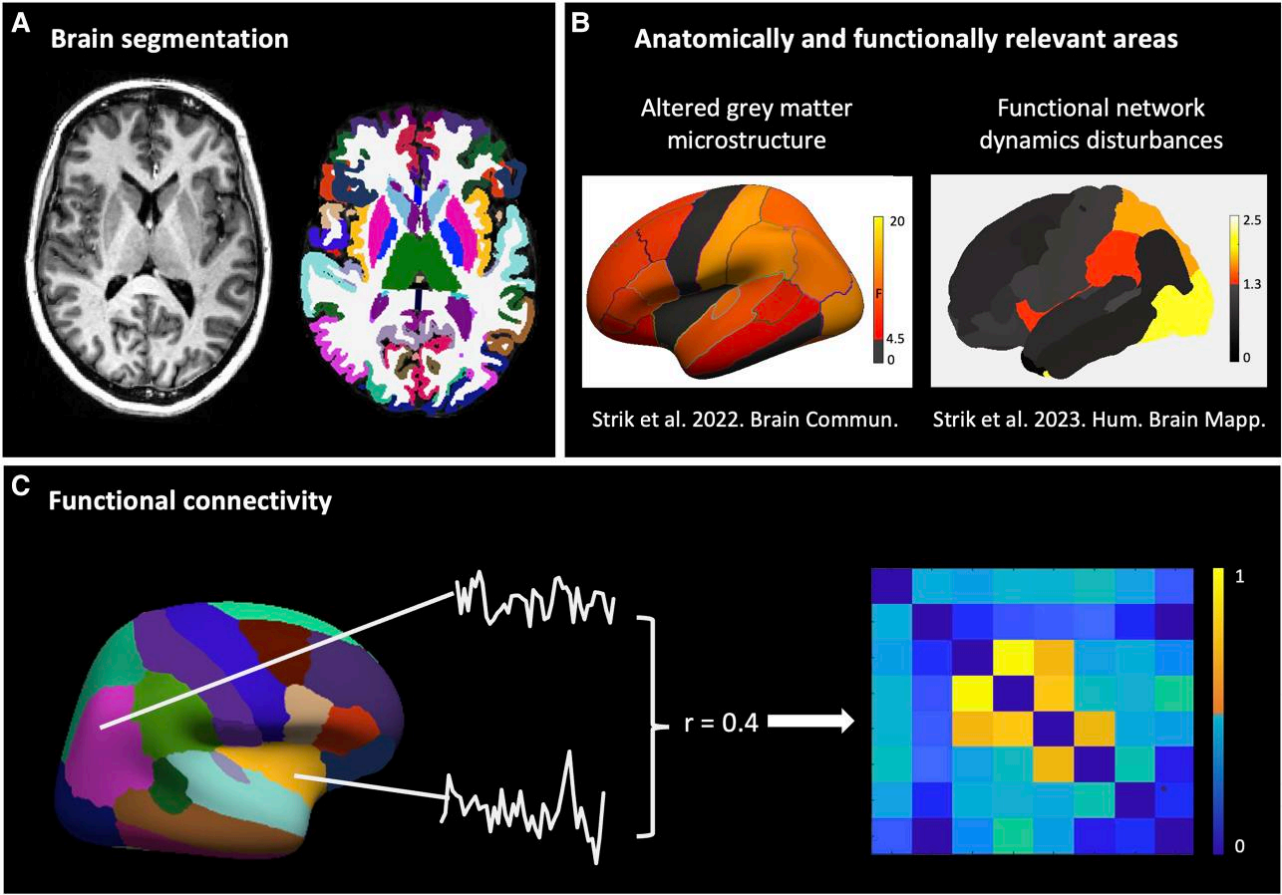
**Functional resting-state processing pipeline**. (**A**) Cortical and subcortical segmentation using the T1-weigthed MP2RAGE image and FreeSurfer. (**B**) Areas that differentiate visual snow syndrome (VSS) patients from healthy individuals (HC), both structurally and functionally, were selected to study further. Structural areas of significant differences are coloured with effect size and functional areas of significant differences with *P* values (−Log10). Images reproduced from Strik *et al*. 2022^7^ and Strik *et al*. 2023.^11^ (**C**) Pre-processed functional MRI signals were extracted from regions of interest and connectivity strength between node pairs was computed using correlation coefficients (**r**), r**e**sulting in an 8 × 8 connectivity matrix for each participant (VSS *n* = 40, HC *n* = 60).

Next, the connectivity and network analyses were performed in MATLAB R2022a (Mathworks, Natick, MA, USA) using customized scripts. For each subject, the functional RS signals were extracted from each ROI. The connectivity between each pair of ROIs was calculated trough pairwise correlations, corrected for average brain connectivity and negative values were set to zero, resulting in an 8 (ROIs)×8 (ROIs)×100 (subjects) matrix (Fig. 1).

### Statistical analyses

All statistical analyses were performed using MATLAB Statistic Toolbox (R2022a, MathWorks, MA, USA). Connectivity between each ROI pair was compared between controls and VSS patients using independent *t*-tests. To study the potential influence of co-morbid migraine, for VSS patients significant MRI metrics were further compared between patients with and without migraine using independent sample *t-*tests. In addition, significant metrics were correlated with ocular motor measures of visual processing and measures of VSS symptoms using Spearman correlations (non-normally distributed data: PS latency (D = 0.243, *P* < 0.001), AS errors (D = 0.201, *P* = 0.002), number of visual snow symptoms (D = 0.157, *P* = 0.025), perceived VSS disruptiveness (D = 0.188, *P* = 0.003), self-rated intensity (D = 0.241, *P* > 0.001).

## Results

### Demographics

Demographics are outlined in Table 1. Patients did not significantly differ from healthy controls on age and sex. Similarly, no differences were observed between patients with and without migraine. Patients were asked about their medical history and medication use, specifically psychiatric and neurological conditions. Out of 40 patients, 25 reported comorbidities, mainly depression (12/40) and anxiety (16/40). Fewer patients reported ADHD (4/40), OCD (2/40) and other conditions, each by one person. Most were not on medication; only one used preventative migraine medication, and five out of 19 took anxiety/depression medication. Seven patients reported past illicit drug use, but none within a week before testing.

**Table 1:** Demographics, visual snow symptoms and self-ratings

Demographics and disease characteristics	Healthy controls	People with VSS	*P*-value	VSS without migraine	VSS with migraine	*P*-value
Participants, n	60	40		17	22	
Age, years	32.24 (8.89)	33.18 (10.08)	0.877	31.55 (9.69)	34.69 (10.54)	0.322
Sex, female/male	35/25	21/19	0.681	44841	44787	0.206
Lifelong VS/sudden onset		15/21		6/9	9/12	
Disease duration, years		20.10 (12.65)		18.59 (12.25)	20.96 (13.33)	0.504
Age of onset if not lifelong, years		23.24 (10.12)		23.11 (10.81)	23.33 (10.07)	0.831
Family history VS, yes/no		1/35		0/15	1/20	
Family history migraine, yes/no		20/16		5/10	15/6	
Visual snow syndrome symptoms						
Visual symptoms^a^ (out of 8)		5.33 (1–8)		5.13 (1–8)	5.48 (3–8)	0.858
After images, yes/no		7/29		11/4	18/3	
Trailing moving objects, yes/no		14/22		11/4	11/10	
Nyctalopia, yes/no		13/23		11/4	12/9	
Photophobia, yes/no		19/17		6/9	11/10	
Floaters, yes/no		4/32		13/2	19/2	
Blue field EP, yes/no		10/26		9/6	17/4	
Self-lightning, yes/no		20/16		7/8	9/12	
Halos, yes/no		9/27		9/6	18/3	
Sensory symptoms^a^ (out of 4)		1.36 (0–4)		1.53 (0–3)	1.24 (0–4)	0.343
Tinnitus, yes/no		13/23		9/6	14/7	
Paraesthesia, yes/no		21/15		8/7	7/14	
Tremors, yes/no		29/7		4/11	3/18	
Dizziness, yes/no		32/4		2/13	2/19	
Migraine with visual aura, yes/no		15/6			15/6	
**Visual snow self-ratings**			*P*-value			*P*-value
Intensity^a^ (1 to 6)		4.03 (1–6)		4.53 (2–6)	3.67 (1–6)	0.020
Disruptiveness^a^ (1 to 7)		3.44 (1–7)		4.00 (1–7)	3.05 (1–5)	0.154
Impact quality of life^a^ (1 to 7)		3.58 (1–7)		3.67 (1–7)	3.52 (1–7)	0.909

Values are presented as means and standard deviations unless stated otherwise. VSS, visual snow syndrome.

^a^Self-rating scores are mean and range.

### Resting-state connectivity alterations in VSS

**Figure 2:**
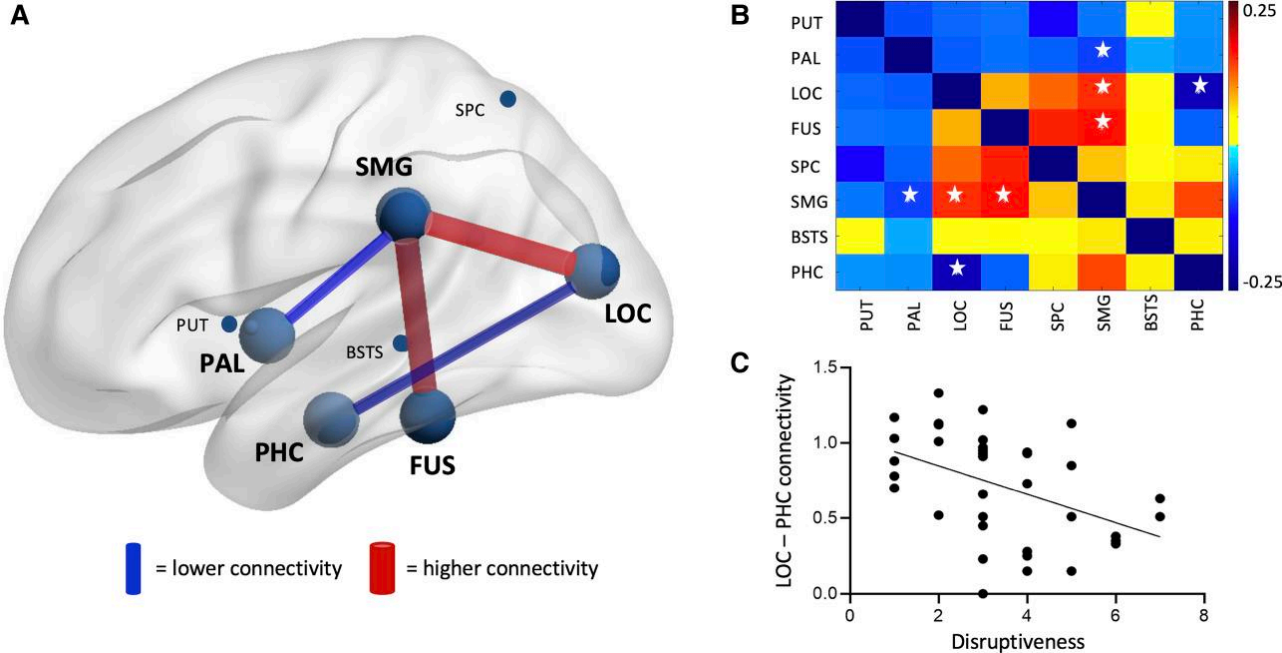
**Altered functional connectivity in visual snow syndrome.** The connectivity differences between visual snow syndrome (VSS, *n* = 40) and healthy controls (HCs, *n* = 60) were assessed using independent *t*-tests and significant differences are visualized in **A** by large circles and lines between those regions and in **B** by a star in the connectivity matrix. Compared to HC, VSS patients showed higher connectivity between the supramarginal gyrus (SMG) and lateral occipital cortex (LOC, *P* = 0.016, *t* = −2.4641) and fusiform (FUS, *P* = 0.007, *t* = −3.5020) and lower connectivity between SMG and pallidum (PAL, *P* = 0.032, *t* = 2.1795), as well as between the parahippocampal gyrus (PHC) and LOC (*P* = 0.007, *t* = 2.7640). (**C**) In VSS, lower connectivity between the LOC and PHC related to higher perceived disruptiveness (*P* = 0.002, *r* = −0.489, Spearman correlation). BSTS, banks of superior temporal sulcus; PUT, putamen; SPC, superior parietal cortex.

Compared with HC, VSS patients demonstrated significantly higher connectivity between the supramarginal gyrus and lateral occipital cortex (*P* = 0.016) and fusiform (*P* = 0.007; Fig. 2A and B). Lower connectivity was found between the supramarginal gyrus and pallidum (*P* = 0.032), as well as between the parahippocampal gyrus and lateral occipital cortex (*P* = 0.007) in VSS patients compared with HC (Fig. 2A and B). Those regions that significantly differed between groups (VSS and HC) did not differ between patients with and without migraine.

### Relation between functional connectivity alterations, oculomotor measures and VSS self-ratings

Significant metrics from the group comparisons were further investigated and the relation with oculomotor measures of visual processing and VSS self-ratings. Within the VSS group, lower connectivity between parahippocampal gyrus and lateral occipital cortex related to higher perceived disruptiveness (*P* = 0.002, *r* = −0.489, Fig. 2C).

## Discussion

This study explored functional connectivity alterations between previously identified brain areas that differentiated VSS from neurological healthy individuals in terms of microstructure as well as functional network characteristics. Where anatomical differences were robust and widespread,^7^ functional changes appear more subtle but are present. Comparing VSS patients to healthy controls uncovered altered functional connectivity between the supramarginal gyrus, fusiform, pallidum, parahippocampal gyrus and lateral occipital cortex. While connectivity between occipital and parietal cortical areas was stronger in individuals with VSS, the identified lower connectivity may imply disconnections between occipital and parietal regions with other parts of the brain, including deep grey matter and temporal cortices. Of these connectivity alterations, lower connectivity between parahippocampal gyrus and lateral occipital cortex related to higher perceived disruptiveness of visual snow.

In individuals with VSS, we observed altered connectivity strength between some of the previously identified regions, centred around the supramarginal gyrus, potentially indicating an important role in VSS. This area that plays a crucial role in processing sensory modalities, potentially associated with frequently reported symptoms in VSS such as tinnitus (ringing sounds) and paraesthesia (‘pins and needles’).^3^ The supramarginal cortex has been indicated by other VSS studies as well.^8,9^ Where we observed stronger connectivity between the supramarginal cortex and the lateral occipital cortex and fusiform, others reported a stronger connectivity with also the primary visual cortex at rest and with middle temporal cortex during visual snow like stimuli.^9^ All these regions are located within the occipital, parietal and temporal lobules and are part of large-scale brain networks involved in vision and attention, indicated to play a role in VSS.^9,10^

The stronger connectivity between the cortical areas found in this study supports the hypothesis of a more rigid network structure in VSS, as suggested previously.^11^ The fundamental aspects of a network organization, network integration and segregation, fluctuated less of time in VSS patients, potentially indicating a more inflexible network topology centred around parietal and occipital areas.^11^ While the mechanisms behind VSS remain uncertain, a more rigid network topology^11^ in combination with the stronger connectivity observed in this study, also aligns with the prominent theory of cortical hyperexcitability.^13-15^ Hyperexcitability has also been indicated by other modalities than MRI. Using magnetoencephalography, the visual cortex showed increased gamma power upon visual stimuli, indicating a hyperexcitable cortex, as well as reduced alpha-gamma phase-amplitude coupling, suggesting an excitation–inhibition imbalance.^15^

In addition to stronger cortical connectivity, we also noted diminished connectivity of the pallidum and putamen with all cortical regions, of which the supramarginal gyrus pallidum connection was found significantly different in VSS compared with controls. Lower connectivity between the deep grey matter and cortical structures in VSS could potentially result in deep grey matter and cortical loop disruptions. Disturbed connectivity of the pallidum may affect the thalamus, a region that regulates and coordinates cortical activity.^16^ Thalamocortical dysrhythmia have been indicated previously to play a role in VSS.^17-19^ While this study did not identify connectivity disturbances specifically related to the thalamus, a more targeted exploration into both the structural and functional network connectivity of the deep grey matter may provide further insights into the underlying mechanisms of VSS.

Even though migraine and visual snow syndrome are distinct neurological disorders, some individuals with VSS reported a history of migraine, and there may be overlapping features, including visual aura-like phenomena. To study the effects of migraine, we compared functional connectivity between VSS patients with and without migraine and observed no differences, suggesting that the functional connectivity alterations found were VSS specific.

As to the clinically importance of these functional connectivity disruptions in VSS, connectivity between lateral occipital cortex and parahippocampal gyrus related to perceived disruptiveness of VSS. The lateral occipital cortex contributes to our ability to make sense of the visual world (visual perception and object recognition), and the parahippocampal gyrus plays a crucial role in spatial memory and navigation and is part of the limbic system, implicated in emotions and emotional memory. The lower the functional connectivity between these two regions, the worse visual snow symptoms were perceived by patients. In particular, the lateral occipital cortex may play an important role in VSS, as the functional dynamics of the lateral occipital cortex also played a role in the ocular motor behaviour typical of VSS.^11^ Shortened prosaccade latencies, previously shown to dissociate individuals with VSS from neurologically healthy individuals,^12^ related to functional efficiency dynamics of the lateral occipital cortex.^11^ Together, these findings, along with earlier research, demonstrate the presence of abnormalities in functional brain network connectivity and their significance in disturbed visual processing and perception of VSS.

Several limitations, challenges and avenues for future research can be acknowledged. Although the sample size is relatively large, conducting future studies with even larger cohorts could enable the assessment of distinct subgroups. Given the cause of VSS remains unknown, studying people with lifelong and a sudden onset of VSS could be of interest. Larger sample sizes may also provide the opportunity to assess the influence of other common comorbidities such as tinnitus and psychiatric symptoms. In addition, distinct network connectivity patterns may be evident and important predictors of conversion of VSS. To study whether the disease changes over time or remains stable, a longitudinal design is needed. We acknowledge the value of pulse or respiration data as confounders and plan to incorporate them in future research to enhance robustness. In addition, the use of a non-validated Likert Scale represents a limitation in this study, highlighting the need for future research to develop and employ validated and objective measures to more accurately assess and quantify VS severity. We did not correct for multiple comparisons to prioritize sensitivity in detecting potential effects, but we acknowledge the risk of false positives and emphasize that the findings should be interpreted cautiously and not overgeneralized. While we studied the brain at rest and observed some subtle functional network connectivity changes, studying the brain while performing an oculomotor task and simultaneously tracking eye movements, could lead to more prominent differences between VSS and healthy controls and insights into visual processing disturbances. Studying the relation between structural and functional alterations or using other imaging techniques such as quantitative MRI including diffusion and susceptibility imaging may provide additional insight into the underlying mechanisms of VSS, what might be explored in future studies.

## Conclusion

In individuals with VSS, we detected changes in functional connectivity strength between brain regions previously identified to be impacted both structurally and functionally. Specifically, we noted stronger functional occipital-parietal connectivity, particularly centred around the supramarginal cortex, but potential disconnections with deep grey matter and temporal cortices. Disruptions in connectivity correlated with a more pronounced perception of VSS symptoms. Taken together, alterations in functional connectivity within these regions imply a deficiency in the brain's ability to function in VSS.

## Data Availability

Data reported in this manuscript can be made available by the corresponding author on reasonable request.
